# Rethinking AI Workflows: Guidelines for Scientific Evaluation in Digital Health Companies

**DOI:** 10.2196/71798

**Published:** 2025-12-04

**Authors:** Kelsey Lynn McAlister, Lee Gonzales, Jennifer Huberty

**Affiliations:** 1 Fit Minded, Inc, 2901 E Greenway Road, PO Box 30271, Phoenix, AZ, 85046, United States, 1 (602) 935-6986; 2 Catalyst AI, Denver, CO, United States

**Keywords:** industry, AI integration, user-centered design, health care delivery, digital health, workflow, scientific evaluation, artificial intelligence

## Abstract

Artificial intelligence (AI) is revolutionizing digital health, driving innovation in care delivery and operational efficiency. Despite its potential, many AI systems fail to meet real-world expectations due to limited evaluation practices that focus narrowly on short-term metrics like efficiency and technical accuracy. Ignoring factors such as usability, trust, transparency, and adaptability hinders AI adoption, scalability, and long-term impact in health care. This paper emphasizes the importance of embedding scientific evaluation as a core operational layer throughout the AI life cycle. We outline practical guidelines for digital health companies to improve AI integration and evaluation, informed by over 35 years of experience in science, the digital health industry, and AI development. It describes a multistep approach, including stakeholder analysis, real-time monitoring, and iterative improvement, that digital health companies can adopt to ensure robust AI integration. Key recommendations include assessing stakeholder needs, designing AI systems that can check its own work, conducting testing to address usability and biases, and ensuring continuous improvement to keep systems user-centered and adaptable. By integrating these guidelines, digital health companies can improve AI reliability, scalability, and trustworthiness, driving better health care delivery and stakeholder alignment.

## Introduction

Digital health companies are increasingly leveraging artificial intelligence (AI) tools to transform care delivery and improve internal operations. AI is being used to develop customer-facing products, such as mental health chatbots and symptom-checking platforms, and to enhance efficiency within organizations, such as accelerating provider documentation workflows [1]. AI adoption has surged globally, with the International Business Machines (IBM) Corporation reporting 35% adoption in 2022 [2], and McKinsey & Company finding this figure had risen to 72% by 2024 [3]. This rapid growth underscores the transformative potential of AI, particularly generative AI (ie, technology that creates new content by learning from existing patterns), which is projected to contribute up to $4.4 trillion in economic value in the coming years [4]. The American Psychological Association named AI as one of the top 10 trends in shaping the field of mental health, recognizing its growing influence [5].

However, AI tools often fail to meet their potential. A recent study highlighted that symptom-checking chatbots frequently provide inaccurate or unhelpful recommendations, eroding user trust and raising patient safety concerns [6]. Similarly, AI-powered transcription tools have been shown to fabricate information or introduce critical errors into clinical documentation, jeopardizing their reliability in real-world settings [7]. Even in research, where AI can support aspects like code automation and study stimuli creation, challenges such as false outputs and breached ethics raise concerns [5]. Additionally, unclear definitions of trust in health care AI contribute to these challenges, hindering ethical and effective translation into practice [8]. These issues underscore the importance of integrating robust, scientifically grounded evaluations into AI tools to enhance their reliability, safety, and effectiveness. The evaluations of AI systems currently tend to prioritize key performance indicators (KPIs) such as efficiency for internal tools and technical performance for customer-facing products to demonstrate return on investment, neglecting essential factors such as usability, transparency, trust, and long-term reliability [9-12]. This fragmented approach to AI evaluation results in several challenges, as internal tools frequently face resistance due to poor design or lack of clarity, and external systems often lose user trust when they fail to perform reliably in real-world contexts [13]. Additionally, the absence of ongoing evaluation and iterative refinement leaves AI systems unable to adapt to evolving needs, which compounds inefficiencies and reduces their long-term impact [13,14]. These gaps undermine the adoption and scalability of AI solutions and jeopardize their potential to drive sustainable change in digital health care.

Sadly, many digital health companies have yet to develop the guidelines and expertise needed to integrate AI effectively and maintain rigorous, ongoing evaluation. Without robust and continuous evaluation built in from the beginning, AI systems risk perpetuating errors, failing to meet stakeholder needs, and losing the trust of end-users. To address the shortcomings of current AI evaluations, including the emphasis on short-term KPIs, neglect of user-centered factors, and the lack of ongoing evaluations, its integration should be approached with a scientific mindset that prioritizes evidence-based methods and continuous learning. A recent clinical-trials-inspired framework emphasizes safety, efficacy, and monitoring, but translating high-level guidance into operational practice remains a challenge for digital health companies [15]. The purpose of this paper is to highlight the importance of embedding scientific evaluation as a core operational layer within AI workflows by providing practical guidelines for decision-makers (eg, C-suite leaders, product and operations leads, clinical directors, and AI implementation managers) in digital health companies. These guidelines are informed by over 35 years of cumulative experience in science, the digital health industry, and AI development. Adopting scientific practices offers digital health companies a pathway to strengthen their approach to AI integration, supporting more reliable and impactful outcomes in digital health care, differentiating themselves from competitors and contributing to revenue generation.

## Guidelines for Integrating and Evaluating AI in Digital Health Companies

### Overview

The following guidelines outline key steps and recommendations to support digital health company leaders in integrating evaluation processes throughout the life cycle of AI systems, enhancing their effectiveness, scalability, and trustworthiness. While conceptually aligned with implementation science frameworks used in AI–such as Consolidated Framework for Implementation Research, which highlights contextual and organizational factors that influence implementation [16], and Proctor’s outcomes, which define success through measures like feasibility and sustainability [17]–these recommendations are tailored to the fast-paced, cross-functional environments in which AI is developed and deployed in digital health.

### Evaluate Stakeholder Needs Before Implementation

Understanding the priorities and needs of stakeholders is a crucial first step to ensure AI systems align with real-world challenges and expectations. Stakeholders may include patients, clinicians, administrators, employees, and app users or consumers, depending on whether the system is designed for internal operations or as an external product. For example, evidence from intensive care settings highlights how involving diverse stakeholders in preimplementation assessments can significantly enhance the success of pilot testing, leading to better AI integration and usability [18]. This approach also facilitates a scientifically grounded evaluation of potential barriers to adoption, such as workflow disruptions or concerns about transparency, that might otherwise hinder long-term success. In addition, co-creation approaches, where stakeholders actively help design and refine AI systems, add value by going beyond traditional consultation [19-21]. These participatory approaches improve alignment with contextual knowledge, increase trust, and promote long-term adoption of AI tools in health care settings [22,23]. By applying scientific evaluation methods at this stage, behavioral and AI scientists, product developers, and operational leaders can systematically identify and address the specific needs and priorities of intended users, guiding the selection and design of AI systems that are both evidence-based and effective in meeting stakeholder requirements.

To assess stakeholder needs effectively, digital health companies should:

- Leverage collaborations and partnerships with industry experts and research scientists. These partnerships can help ensure that the AI system aligns with scientific standards while also remaining feasible for implementation within the company.
- Conduct qualitative and quantitative assessments with end-users to understand expectations, pain points, desired outcomes, and what AI platforms they may already use. Research and user experience teams can spearhead these efforts, leveraging a human-centered approach to ensure the system aligns with real-world needs and user priorities [24,25]. Qualitative methods offer in-depth insights, while quantitative approaches help capture broader trends across diverse user groups. For example, a digital health company could interview clinicians to uncover documentation challenges, then run a survey to assess anticipated usability, perceived efficiency, and readiness to adopt an AI tool.
- Use co-creation strategies, such as co-design workshops or participatory prototyping, to allow stakeholders to directly influence system functionality, content, and workflows. These methods surface context-specific needs that traditional assessments may miss and help improve usability, trust, and alignment with end-user expectations.
- Develop user personas and journey maps to understand how the AI system fits into existing workflows or end-user experiences. This approach can help teams visualize user interactions, surface potential friction points, and inform refinements that support usability and integration, especially when combined with direct stakeholder input gathered through participatory design activities.

### Design AI Systems That Check Its Own Work

To ensure that AI systems are robust, effective, and user-centered throughout their life cycle, digital health companies should embed scientific evaluation mechanisms directly into their design. However, many companies currently underinvest in rigorous evaluation processes, leading to inconsistent progress, flawed AI tools that fail to meet business objectives, canceled projects, and wasted resources [26]. AI evaluations should balance traditional KPIs, such as accuracy and efficiency, with metrics that allow the system to monitor and reflect on user experience, trust, usability, and satisfaction. By enabling AI tools to “check their own work,” companies can create systems that not only meet company goals but also foster user trust and adoption—key factors for achieving sustained impact and scalability in health care settings. This includes designing systems that can detect uncertainty, surface potential issues, and escalate to human input when appropriate. Companies should consider the cross-functional work of teams such as engineering, technology, and science to ensure appropriate design, implementation, and evaluation of AI systems that align with stakeholder needs and operational goals.

In particular, digital health companies should integrate human-in-the-loop (HITL) methodologies, an approach that embeds human judgment into the training, validation, and deployment phases of AI tools. This enables teams to guide model development, intervene during deployment, and refine outputs in real-time, improving adaptability, safety, and trustworthiness [27,28]. HITL is distinct from broader governance or post-hoc audits in that it provides direct, real-time oversight within system workflows. This is especially important in clinical and behavioral health contexts, where ethical and contextual judgment cannot be fully automated.

Several scientific frameworks have been developed to evaluate AI tools, offering valuable guidance on embedding evaluation into the design process. Frameworks such as Standard Protocol Items: Recommendations for Interventional Trials-AI (SPIRIT-AI; [29]) and Consolidated Standards of Reporting Trials–AI (CONSORT-AI; [30]) focus on building transparency, trust, and rigor during the design and reporting phases of clinical trials for AI. While these frameworks emphasize preimplementation evaluation, others, such as Translational Evaluation of Healthcare AI (TEHAI; [31]) and Explainable AI (XAI; [32]), address specific aspects like performance, safety, ethical considerations, and user trust.

While these frameworks provide a strong foundation, they often focus on discrete stages of evaluation and may not fully incorporate HITL approaches that enable continuous input and oversight throughout the AI life cycle. Digital health companies must go further by embedding evaluation mechanisms into workflows to ensure continuous monitoring and improvement. Without such mechanisms, teams risk uneven progress, flawed implementations, and ultimately, AI tools that fail to meet stakeholder needs or achieve business goals [26].

When building AI systems that can monitor themselves, digital health companies should:

- Prioritize early investment in AI evaluation to build a strong foundation for assessing effectiveness throughout the AI tool’s life cycle. This approach ensures potential challenges are proactively addressed, which supports smoother implementation and long-term adaptability. This may include consulting with behavioral or AI scientists to design evidence-based evaluation methods, identify potential biases, and refine AI system performance to align better with real-world needs.
- Consider existing scientific frameworks as a foundation for designing AI tools with transparency and rigor, while adapting them to include mechanisms for ongoing, real-world evaluation that captures both technical performance and comprehensive user experience metrics.
- Develop automated tools for ongoing evaluation that track metrics aligned with both user priorities and business objectives, such as technical accuracy, error rates, user satisfaction, and productivity. These evaluations streamline development by concentrating efforts on critical areas, increasing the likelihood of deploying AI systems that effectively meet organizational goals and end-user needs [26]. For example, automated tools could monitor user interactions on a mental health platform, such as response times, task completion rates, and drop-off points, allowing product teams and behavioral or AI scientists to identify areas for improvement and enhance user experience.
- Establish feedback loops that allow for end-users to provide feedback in real time, ensuring their perceptions are consistently captured and integrated into system updates.
- Embed HITL components such as human review panels, clinician-in-the-loop decision support, or structured escalation processes that ensure human judgment is available at key junctures. HITL differs from general human oversight or feedback mechanisms in that it places human judgment directly within the AI system’s workflow, enabling real-time intervention to correct system drift, mitigate error propagation, and uphold ethical safeguards.
- Incorporate routine bias audits into evaluation workflows to assess whether the AI system performs equitably across user subgroups. This is particularly important in health settings where automated systems can unintentionally amplify disparities, especially among low-prevalence or underserved populations [33,34]. Regularly reviewing model outputs by demographic characteristics and edge cases can help teams identify and mitigate bias early in the deployment cycle.
- Establish human oversight to ensure accountability, mitigate potential biases, and validate performance. This includes establishing a scientific AI evaluation leader or multidisciplinary review teams to regularly assess the system’s outputs, identify blind spots in automated evaluations, and ensure alignment with company goals and user needs. Human input is critical for addressing nuances and ethical considerations that AI alone may overlook, ensuring the system’s outputs remain contextually appropriate and trustworthy. Oversight reinforces governance and long-term trust, complementing the real-time, embedded nature of HITL.

### Testing and Refinement Before Implementation

AI systems should undergo beta, feasibility, and pilot testing, which involves the collaboration of research (ie, behavioral and AI scientists), user experience, product, engineering, and operations teams, to ensure they are ready for real-world implementation. These phases allow opportunity to identify potential issues related to usability, performance, and integration within real-world workflows before full implementation. For example, beta testing helps gather quick feedback from real-world users to refine usability and make early improvements. Feasibility can be used to evaluate resource requirements and alignment with the business goals of the company, ensuring practical deployment and sustainability. Pilot testing can also be used to further refine the AI tool and assess initial outcomes for viability. For instance, pilot testing has been essential in improving the intuitiveness of health care chatbots [35,36].

To effectively test and refine before implementation, digital health companies should:

- Conduct feasibility tests early to assess whether the AI tool aligns with business objectives, technical infrastructure, and resource availability. This ensures the AI is viable and positioned for successful implementation.
- Engage diverse stakeholders, such as clinicians, administrators, and end-users during testing and refinement to gather comprehensive feedback. For example, when testing an AI-powered clinical decision support tool, a company could engage physicians to ensure recommendations align with clinical guidelines, administrators to assess integration with existing electronic health record systems, and nurses to evaluate usability and workflow compatibility.
- Iterate, based on findings, by using the feedback from these testing stages to refine the AI system, addressing issues such as workflow integration and potential user resistance. Structured reviewer input or user flagging mechanisms, when embedded into the system’s operation, can function as HITL approaches that support more responsive and ethical refinement. AI scientists and operations teams should work closely with behavioral scientists to ensure the system evolves based on real-world insights.

### Implement With Real-Time Monitoring and Data Collection

Implementing an AI tool is an important opportunity for digital health companies to gather actionable insights about how they perform in real-world settings. Real-time monitoring and data collection allow companies to identify emerging issues, refine workflows, and validate that AI tools meet both technical and user-centered expectations. For example, LinkedIn leveraged a deep-learning-based monitoring system to track the health of its AI models, identifying issues in real-time to improve business outcomes [37]. This proactive approach supports scalability and long-term adoption by addressing challenges early in deployment. Engineering, operations, and research (eg, behavioral and AI scientists and data analysts) should collaborate to establish monitoring systems and analyze findings.

To implement real-time monitoring and data collection effectively, digital health companies should:

- Deploy automated, self-check monitoring systems to continuously track both traditional KPIs (eg, accuracy, response times, and error rates) and user experience metrics (eg, task completion rates, interaction frequency, and perceived usability). Leverage the evaluation mechanisms embedded during the AI system’s design.
- Analyze incoming data to systematically identify patterns or recurring issues that may impact the AI’s performance or user engagement. Structured analyses, conducted by the company’s data analysts and behavioral scientists, help prioritize areas for improvement and ensure resources are allocated effectively. For example, by analyzing user interaction patterns, a company might find that users tend to leave an AI-powered chat when provided with lengthy responses, prompting the need to shorten message length to improve engagement.
- Implement strategic refinements based on monitored insights to address significant challenges or adapt the AI system to evolving user needs and company priorities. Postlaunch updates should be carefully planned and aligned with long-term goals. Where appropriate, HITL mechanisms can support these refinements by enabling human input in ambiguous, high-stakes, or ethically sensitive situations.

### Continue Evaluation and Iterative Improvement After Implementation

Once an AI system is implemented, ongoing evaluation becomes essential to ensure it continues to meet company goals and user expectations. AI tools often experience performance degradation over time as changes in usage patterns, data inputs, workflows, user needs, and external factors (eg, regulatory changes, updates to clinical guidelines) require them to adapt to maintain their effectiveness and relevance. For example, generative AI models (eg, ChatGPT) present unique challenges due to their inherent randomness, making repeated evaluations essential to ensure reliable performance [38]. Additionally, the dynamic nature of AI, particularly generative AI, requires digital health companies to continuously adopt and adapt to rapidly improving models with enhanced capabilities and significant cost fluctuations. Recent benchmarking data from Epoch shows that once models reach certain levels of computing power, they experience significant jumps in performance on tasks [39]. Furthermore, when GPT-4 was initially released in March 2023, it cost US $36 per million tokens (ie, units of text used to process input and generate output), but by late 2024, this price had dropped to just $0.25 per million tokens—a staggering 99% reduction [40]. This sharp drop in cost highlights how quickly AI technology evolves, making advanced tools more affordable over time. For digital health companies, this means they must regularly evaluate whether adopting updated models is both practical and beneficial, ensuring they use the most effective and cost-efficient solutions while staying aligned with their goals and user needs. Product teams, behavioral and AI scientists, and operation specialists should collaborate to monitor performance, gather user feedback, and adapt systems to evolving needs and guidelines.

To continuously evaluate and improve AI systems, digital health companies should:

- Conduct regular audits to assess technical metrics, such as accuracy and reliability, alongside user experience metrics, including satisfaction and usability. These audits help identify whether the system is meeting its intended objectives and uncover opportunities for optimization. They should also maintain broader human oversight (beyond HITL mechanisms), which could include a scientific AI evaluation leader and/or a multidisciplinary team
- Incorporate usability testing as a continuous process to regularly identify pain points and opportunities for improvement among diverse user groups. Regularly engaging with end-users ensures that the system adapts to their evolving needs and remains intuitive and efficient. For example, ongoing usability testing for an AI-driven mental health platform could involve observing end-users as they navigate key features, such as finding a therapist or accessing self-help tools, to identify usability challenges and inform iterative design improvements.
- Prioritize publishing and reporting on AI performance, user experiences, and trust-building metrics throughout AI integration (ie, from beta testing to post-launch). Reporting on technical metrics alongside user-focused insights offers a holistic view of AI system effectiveness. For example, companies can conduct retrospective analyses of de-identified conversation content and usage patterns to identify trends and gaps, guiding future improvements. While peer-reviewed outputs are valuable, resource-constrained teams may benefit from alternative dissemination methods, such as implementation briefs, open-access case reports, webinars, or practice-based repositories, that enable rapid, practical knowledge-sharing. By sharing these findings, companies contribute to greater accountability, advance innovation, and guide the development of AI tools that meet user and company needs.
- Maintain human oversight that could include a scientific AI evaluation leader or a multidisciplinary team of technical, clinical, and operational experts. Human oversight is needed to ensure that AI systems can continuously adapt to new data, address unforeseen issues, and uphold ethical and performance standards in dynamic health care settings.

## Conclusions

The integration of AI into digital health presents a transformative opportunity to enhance care delivery, optimize operations, and improve patient outcomes. However, its success hinges on a commitment to continuous, scientifically grounded evaluation. Scientific evaluation is not just a checkpoint—it is an operational layer that should be embedded into workflows to ensure trust, scalability, and measurable impact. While not developed through a formal consensus process or systematic review, the guidelines outlined in this paper are informed by over 35 years of cumulative experience across science, the digital health industry, and AI development. They advocate for incorporating scientific evaluation processes that balance technical performance with user-centered metrics, enabling digital health companies to ensure their AI tools remain effective, adaptable, and trustworthy over time. This approach may enhance the reliability and scalability of AI systems and drive revenue growth by improving user satisfaction, increasing adoption rates, and streamlining operations. Achieving these outcomes requires cross-functional collaboration between behavioral and AI scientists, data analysts, product teams, engineers, and operations staff. Together, these teams can ensure AI solutions are aligned with business objectives, meet stakeholder needs, and deliver meaningful, scalable impact in digital health care. A key future direction is to formally build on these recommendations through a structured, cross-disciplinary consensus process.

## Abbreviations

AI: artificial intelligence
CONSORT-AI: Consolidated Standards of Reporting Trials–AI
HITL: human-in-the-loop
KPI: key performance indicator
SPIRIT-AI: Standard Protocol Items: Recommendations for Interventional Trials-AI
TEHAI: Translational Evaluation of Healthcare AI
XAI: Explainable AI
